# Investigating the relationship between thyroid replacement therapy goals status and quality of life in patients with primary hypothyroidism

**DOI:** 10.1007/s12020-026-04642-3

**Published:** 2026-05-02

**Authors:** Ayşe Kubat Üzüm, Zeynel Abidin Sayiner, Kader Uğur, Sadettin Öztürk, Güzide Gonca Örük, Ayten Eraydın, Havva Sezer, Abbas Ali Tam, Fatma Nur Korkmaz, Çiğdem Tura Bahadır, Ziynet Alphan Üç, Filiz Ekşi Haydardedeoğlu, Mehtap Evran, Barış Karagün, Emre Saygılı, Dilek Tüzün, Züleyha Karaca, Hülya Hacışahinoğulları, Gülhan Akbaba, Meriç Coşkun, Tayfun Garip, Müge Özsan Yılmaz, Eren Gürkan, Gökçen Ünal Kocabaş, Nurdan Gül, Mustafa Koçak, Ahmet Numan Demir, Güven Barış Cansu, Mustafa Kulaksızoğlu, Mustafa Ünübol, Murat Çalapkulu, Mehmet Muhittin Yalçın, Füsun Baloş Törüner, Mustafa Sait Gönen, Mehmet Erdoğan, Dilek Yazıcı, Bekir Çakır, Murat Faik Erdoğan, Ersin Akarsu

**Affiliations:** 1 Department of Internal Medicine, Division of Endocrinology and Metabolism, Istanbul University School of Medicine, Istanbul, Turkey; 2 Department of Internal Medicine, Division of Endocrinology and Metabolism, Gaziantep University School of Medicine, Gaziantep, Turkey; 3 Department of Internal Medicine, Division of Endocrinology and Metabolism, Sanko University School of Medicine, Gaziantep, Turkey; 4 Department of Internal Medicine, Division of Endocrinology and Metabolism, Fırat University School of Medicine, Elâzığ, Turkey; 5 Department of Internal Medicine, Division of Endocrinology and Metabolism, Gaziantep State Hospital, Gaziantep, Turkey; 6 Department of Internal Medicine, Division of Endocrinology and Metabolism, İzmir Atatürk Training and Research Hospital, İzmir, Turkey; 7 Department of Internal Medicine, Division of Endocrinology and Metabolism, Pamukkale University School of Medicine, Denizli, Turkey; 8 Department of Internal Medicine, Division of Endocrinology and Metabolism, Koç University School of Medicine , Istanbul, Turkey; 9 University of Health Sciences, Diskapi Yildirim Beyazit Training and Research Hospital, Department of Endocrinology and Metabolism, Yıldırım Beyazit University, Ankara, Turkey; 10 Department of Internal Medicine, Division of Endocrinology and Metabolism, Ankara University School of Medicine, Ankara, Turkey; 11 Department of Internal Medicine, Division of Endocrinology and Metabolism, Amasya University, Sabuncuoglu Serefeddin Education and Research Hospital, Amasya, Turkey; 12 Department of Internal Medicine, Division of Endocrinology and Metabolism, Usak University School of Medicine, Usak, Turkey; 13 Department of Internal Medicine, Division of Endocrinology and Metabolism, Başkent University School of Medicine, Adana, Turkey; 14 Department of Internal Medicine, Division of Endocrinology and Metabolism, Çukurova University School of Medicine, Adana, Turkey; 15 Department of Internal Medicine, Division of Endocrinology and Metabolism, Çanakkale Onsekiz Mart University School of Medicine, Çanakkale, Turkey; 16 Department of Internal Medicine, Division of Endocrinology and Metabolism, Kahramanmaraş Sütçü İmam University School of Medicine, Kahramanmaraş, Turkey; 17 Department of Internal Medicine, Division of Endocrinology and Metabolism, Erciyes University School of Medicine, Kayseri, Turkey; 18 Department of Internal Medicine, Division of Endocrinology and Metabolism, Muğla Sıtkı Koçman University School of Medicine, Muğla, Turkey; 19 Department of Internal Medicine, Division of Endocrinology and Metabolism, Gazi University School of Medicine, Ankara, Turkey; 20 Department of Internal Medicine, Division of Endocrinology and Metabolism, Istanbul Medipol University School of Medicine, Istanbul, Turkey; 21 Department of Internal Medicine, Division of Endocrinology and Metabolism, Mustafa Kemal University School of Medicine, Hatay, Turkey; 22 Department of Internal Medicine, Division of Endocrinology and Metabolism, Ege University, İzmir, Turkey; 23 Department of Internal Medicine, Division of Endocrinology and Metabolism, Karadeniz Technical University, Trabzon, Turkey; 24 Department of Internal Medicine, Division of Endocrinology and Metabolism, Istanbul University Cerrahpaşa Faculty of Medicine, Istanbul, Turkey; 25 Department of Internal Medicine, Division of Endocrinology and Metabolism, Kütahya Health Sciences University, Kütahya, Turkey; 26 Department of Internal Medicine, Division of Endocrinology and Metabolism, Necmettin Erbakan University Meram School of Medicine, Konya, Turkey; 27 Department of Internal Medicine, Division of Endocrinology and Metabolism, Adnan Menderes University School of Medicine, Aydın, Turkey

**Keywords:** Euthyroidism, Hypothyroidism, Levothyroxine, Patient reported outcome measures, Quality of Life

## Abstract

**Background and objective:**

The relationship between quality of life (QoL) and thyroid stimulating hormone (TSH) levels in patients receiving levothyroxine (LT4) therapy is unclear. Our aim was to determine the efficacy of replacement therapy and the relationship between TSH concentration and QoL in patients diagnosed with primary hypothyroidism and receiving LT4 replacement therapy.

**Methods:**

This is a national retrospective cross-sectional study. Demographic information, a questionnaire for diagnosis and management of thyroid diseases and Thyroid related Patient Reported Outcome (ThyPRO ) and EQ-5D questionnaires to assess quality of life were used to collect data. The study included retrospective 3 year follow up data.

**Results:**

Our study included 1750 patients (90.3% female, 9.7% male) from 26 centers with a mean age of 47.8 ± 12.6 years (18–88) and a body mass index of 28.84 ± 5.89 kg/m2. Patients had been receiving levothyroxine replacement for 10.23 ± 7.03 (3–55) years. The mean number of visits during the three-year follow-up period was 6.34 ± 2.78 [1–22]. A total of 188 patients (10.7%) had never reached the specific TSH target. The target TSH value was reached < 3 times (*n* = 715; 40.8%), 3–5 times (*n* = 775; 44.2%), and ≥ 6 times (*n* = 260; 14.8%). According to the ThyPRO questionnaire, patients who achieved their treatment target fewer than 3 times had higher (worse thyroid related quality of life) scores in several domains, including goiter symptoms, hyper-/hypothyroid symptoms, fatigue, cognitive complaints, anxiety, depression, and impairments in social, sexual, and daily functioning. In contrast, these patients had lower EQ-5D scores, indicating poorer overall health-related quality of life.

**Conclusion:**

In hypothyroidism replacement therapy, it is crucial to establish an individualized TSH target, and the patient’s cognitive functions and quality of life are positively impacted by the attainment of euthyroidism.

## Introduction

Over 10% of the adult population is affected by thyroid diseases. The prevalence of these conditions can vary significantly among various geographic regions. The general population’s prevalence of overt and subclinical hypothyroidism is estimated to be between 3% and 7% [1]. In Europe, the prevalence of overt hypothyroidism in the general population ranges from 0.2% to 5.3%. Women have a hypothyroidism prevalence that is approximately ten times greater than that of men [2]. Hashimoto’s autoimmune disease is the primary cause of the condition. It can also occur because of radioactive iodine treatment for Graves’ disease or following thyroid surgery. Levothyroxine (LT4) is acknowledged as the primary treatment for hypothyroidism and has become an effective treatment option [3]. It is estimated that between 30% and 50% of patients who are treated with LT4 are either undertreated or overtreated. The reasons for this low rate include the presence of comorbid diseases, nutritional errors, drug compliance problems, drug interactions, and malabsorption [4]. The primary objectives of hypothyroidism treatment are to normalize thyroid stimulating hormone (TSH) levels, enhance symptoms, and enhance quality of life. The quality of life of individuals is adversely affected by both under and over treatment, which can result in a variety of health issues [5]. The quality of life of patients who are undergoing thyroid hormone replacement therapy can be evaluated using specific measurement tools and scales. It is underscored that there is a lack of research on the efficacy of levothyroxine treatment in the general population [6]. It is obvious that optimal TSH levels are crucial. Nevertheless, certain patients with normalized TSH levels continue to experience symptoms that are indicative of hypothyroidism, which has a detrimental effect on their overall quality of life [7]. Achieving an optimal TSH level through levothyroxine therapy enhances patients’ symptoms and overall health status, which are also essential variables influencing their quality of life. Therefore, instead of exclusively focusing on TSH levels, employing Thyroid-related Patient-Reported Outcome (ThyPRO), a thyroid-specific tool for assessing symptom management, may offer additional benefits in assessing patients’ quality of life [8]. The literature reveals a scarcity of studies concerning the influence of abnormal TSH values on patients’ quality of life during the follow-up period.

In this study, we evaluated the rates of achieving target TSH concentrations with levothyroxine replacement therapy in patients with primary hypothyroidism receiving levothyroxine replacement therapy who had at least three years of retrospective data available, the rate of continuing to experience hypothyroidism symptoms despite euthyroidism, and medication adherence among patients. We also aimed to investigate the relationship between TSH concentration at the last visit and the ThyPRO survey and the Quality of Life Questionnaire (Eq. 5D).

## Materials and methods

### Design and ethics

This study was designed to include all geographical regions (7 regions/18 cities) in Türkiye. This is an observational study utilizing a questionnaire administered to patients with hypothyroidism across 26 health centers. The protocol was submitted to and approved by the Medical Ethics Committee of Istanbul University, Istanbul Faculty of Medicine (Protocol No:29 Date: 20/11/2020). All participants were apprised of the study’s purpose and nature. They willingly filled out the questionnaire and thereby agreed to participate in the study. All patients underwent measurements of height and weight. ThyPRO and Eq. 5D questionnaires were completed.

### Patient selection

The eligibility criteria required individuals to be a minimum of 21 years old. The patient has been monitored at the same center for more than 36 months, has a diagnosis of primary hypothyroidism, and is presently undergoing levothyroxine replacement therapy. The exclusion criteria included undergoing suppression therapy for thyroid cancer; presence of a disease associated with absorption defects (i.e. inflammatory bowel disease, celiac disease or having undergone bariatric surgery), presence of pregnancy or lactation period, psychiatric disorder that could impair compliance and presence of hypoparathyroidism.

### Reference ranges of thyroid parameters

Thyroid disease guidelines recognize thyroid stimulating hormone (TSH), free T4 (fT4), and free T3 (fT3) levels as benchmarks for normal ranges. The target TSH was accepted as 0.4–2.5 mU/L for young and middle-aged individuals without comorbidities and up to 4.0 mU/L for those with comorbidities. For individuals aged 65 to 80 years, the TSH target was 3 to 6 mU/L and ≤ 10 mU/L for those over 80 years old. Thyroid peroxidase antibody (TPO Ab) tests were classified as negative for results ≤ 100 IE/mL and positive for results exceeding 100 IE/mL [9].

### ThyPRO questionnaire

We assessed quality of life utilizing the validated thyroid-specific instrument ThyPRO. A study on the validity and reliability of the Turkish translation of the ThyPRO questionnaire has been conducted [10]. The ThyPRO quality of life domains relevant to patients include illness symptoms, fatigue, vitality, memory and concentration, irritability and mental intensity/nervousness, depressive feelings, emotional sensitivity, social and daily functioning, and symptoms associated with hypothyroidism. The ThyPRO survey consists of 84 items summarized in 13 scales as well as a single item measuring overall impact of thyroid disease on quality of life. The ThyPRO questionnaire does not yield a total overall score. Each question utilizes a five-point Likert scale for measurement (0 = not at all), 1 denotes minimal quantity, 2 equals a small amount, 3 signifies a slight increase, 4 = significantly. The average score of questions in a scale is divided by four and multiplied by 100 to yield 0–100 scales, with higher scores indicating worse health status [11]. We compute a distinct score for each scale (ANNEX-8).

### EQ-5D quality of life scale

The EuroQol Group developed the EQ-5D as a standardized instrument for assessing health-related quality of life. It can serve as a simple and universal questionnaire for clinical and economic evaluations, as well as population health surveys. EQ-5D is a patient-reported outcome measure enabling individuals to autonomously complete a questionnaire to convey their current health status and monitor changes over time. The descriptive system consists of five dimensions: health profile, mobility, self-care, social life, pain, and psychological status. It serves as an indicator of the individual’s health status at that moment. The EuroQol Group’s Version oversees the translation of the EQ-5D into various languages [13].

We categorized the patients according to the number of visits that reached the individualized target. Group 1: patients who achieved the target 0–2 times (*n* = 715; 40.8%), group 2: patients who achieved the target TSH value for 3–5 times (*n* = 775; 44.2%), group 3: patients who achieved the target TSH value for ≥ 5 (*n* = 260; 14.8%). Also, we evaluated the mean TSH concentration of the patients according to their decades.

### Statistical analysis

All statistical analyses were performed using SPSS version 28 (IBM Corp., Armonk, NY, USA). Continuous variables were summarized as mean±standard deviation or median, and categorical variables were expressed as numbers and percentages. The distribution of continuous variables was assessed using the Kolmogorov Smirnov and Shapiro Wilk tests. Comparisons between groups were performed using the Student’s t-test or one way ANOVA for normally distributed variables and the Mann Whitney U or Kruskal Wallis tests for non-parametric distributed variables. Categorical data were compared using the chi-square test or Fisher’s exact test when required. Associations between continuous variables (dose, disease duration, BMI, and quality-of-life scores) and the number of TSH target achieving visits were evaluated using Pearson or Spearman correlation analyses. A p-value < 0.05 was considered statistically significant throughout the analyses.

## Results

The average age of the patients was 47.8 ± 12.6 years (21–88 years). Among the 1750 participants, 1582 (90.3%) were female and 168 (9.7%) were male. The mean duration of levothyroxine therapy for hypothyroidism was 10.23 ± 7.03 years (3–55 years). The mean body mass index (BMI) of the patients was 28.84 ± 5.89 kg/m². The average number of visits attended by patients during the follow-up period was 6.34 ± 2.78 visits. The most common causes of hypothyroidism were Hashimoto thyroiditis (*n* = 1200; 68.6%), thyroid surgery (*n* = 467; 26.7%), and radioactive iodine treatment (*n* = 58; 3.3%). Table 1 presents the demographic and clinical characteristics of the participants.

**Table 1:** The participants’ demographic and clinical characteristics

	Parameters	*n*	%
**Gender**	Female Male	1582 168	90.3 9.7
**Family history of hypothyroidism**	Yes No	900 850	51.4 48.6
**Eduactional status**	Illiterate Primary School Middle School High School University	86 597 193 408 465	4.9 34.1 11.0 23.3 26.6
**Working status**	Not working 08:00–18:00 working hours Shifts	1282 433 40	73.3 24.7 2.3
**Marital status**	Single Married	316 1434	18.1 81.9
**Etiology of hypothyroidism**	Hashimoto’s thyroiditis Surgery RAI treatment Other (Subacute thyroiditis. congenital, radiotherapy on neck region, drugs)	1200 467 58 25	68.6 26.7 3.3 1.4
**Symptoms**	Amnesia Skin dryness Weight gain Muscle cramps, pain, numbness Joint pain Chills Constipation Edema. Weakness, fatigue Perception difficulties Depression Hoarseness Decreased hearing Menstrual irregularity	1011 901 874 832 807 786 652 619 520 505 480 407 355 302	57.8 51.5 49.9 47.5 46.1 51.5 37.3 44.9 29.7 28.9 27.4 23.3 20.3 19.1

The patients were categorized based on their age in decades. The overall study group received an average daily dose of 97.32 ± 43.89 µg of L-T4. The daily L-T4 dose (µg/day) was 89.88 ± 42.6 µg in Hashimoto thyroiditis group, 117.03 ± 52.7 µg in total thyroidectomy group and 108.91 ± 48.34 µg in radioiodine treatment group. The lowest daily dose was found in Hashimoto thyroiditis group; the average daily dose was lower in the Hashimoto thyroiditis group compared to the total thyroidectomy group (89.88 ± 42.6 µg vs. 117.03 ± 52.7 µg; *p* < 0.01) of L-T4. Patients (*n* = 179) with a TSH concentration < 0.5 mU/L at the most recent visit had a longer duration of hypothyroidism compared with those whose TSH levels were within the reference range (0.5–4 mU/L). Despite receiving lower daily and weekly levothyroxine doses and having lower body weight, these patients achieved the individualized target TSH less frequently during the follow-up period, suggesting a tendency toward overtreatment and greater variability in TSH control. The duration of hypothyroidism was shorter in patients with Hashimoto’s thyroiditis compared to the total thyroidectomy group (9.7 ± 6.46 vs. 11.64 ± 8.18 years; *p* < 0.001), whereas the difference between the Hashimoto’s thyroiditis and radioactive iodine groups was not significant (9.7 ± 6.46 vs. 10.10 ± 6.96 years; *p* > 0.05). Weight, weekly levothyroxine doses were significantly lower (Table 2).

**Table 2:** Comparison of the hypothyroidism etiologies according to clinical parameters

	Hashimoto’s thyroiditis (*n* = 1225)	Thyroidectomy (*n* = 467)	Radioactive iodine treatment (*n* = 58)
**Hypothyroidism duration (years)**	9.70 ± 6.45^a.b^	11.64 ± 8.18	10.10 ± 6.96
**Age (years)**	46.38 ± 12.84^a.b^	51.11 ± 11.49	52.14 ± 12.7
**Weight (Kilogram)**	73.85 ± 15.17^a.b^	78.13 ± 15.69	77.86 ± 13.53
**Body mass index (kg/m** ^**2**^ **)**	28.84 ± 5.80^a.b^	29.01 ± 6.16	27.25 ± 5.32
**Daily dose (mcg)**	89.88 ± 42.60^a.b^	117.03 ± 52.7	108.91 ± 48.34
**Weekly dose (mcg)**	625.28 ± 300.24^a.b^	809.42 ± 283.62	776.17 ± 343.13

^a^ Comparison of patients with Hashimoto’s thyroiditis and thyroidectomy: *p* < 0.001

^b^ Hashimoto’s thyroiditis and Radioactive iodine treatment: *p* < 0.01

In the 36 months follow-up period, the average number of visits was 6.34 ± 2.78 [1–22]. The individual TSH target had never been achieved by 188 patients (10.7%). The educational status of the patients who participated in the study was as follows: 4.9% were illiterate, 34.1% were primary school graduates, 11% were secondary school graduates, 23.3% were high school graduates, and 26.6% were university graduates. Most patients were unemployed (73.3%), while 27.3% were employed in shift-based jobs. Regarding treatment patterns, 49.4% of patients had variable daily doses, whereas 50.6% received consistent dosing. A total of 94.1% of the patients were attentive to the timing of their medication intake. A total of 33% of the patients reported forgetting to take levothyroxine once or twice per week. The mean TSH concentration of patients aged 21–29 years was 4.76 ± 8.48 mU/L (*n* = 129), those aged 30–39 was 3.47 ± 4.65 mU/L (*n* = 267), 40–49 years (*n* = 469) was 3.33 ± 4.85, 50–59 years (*n* = 434) was 3.86 ± 10.08 mU/L, 60–69 years (*n* = 228) was 3.54 ± 9.69 mU/L, 70–79 years (*n* = 70) was 3.09 ± 3.05 mU/L and those ≥ 80 years (*n* = 3) was 1.93 ± 0.57 mU/L (Table 3).

**Table 3:** Evaluation of patients’ medication compliance and TSH target

	*n*	%		*n*	%
**Daily doses** Same dose Different dose Consecutive days Distributed over 7 days	886 864 706 158	50.6 49.4 81.7 18.2	Forgetting to take the drug No Yes	1173 577	67.0 33.0
**Careful timing of drug intake** Yes No	1647 102	94.18 5.82	**Timing of taking the medicine** <30 min before breakfast 30 min before breakfast 30–60 min before breakfast 60 min before breakfast After breakfast	414 497 424 362 12	23.7 28.4 24.2 20.7 0.7
**Continuously used medication** Yes No	1004 746	57.6 42.6	**Continuously used medication** Proton pump inhibitors Oral iron preperation Oral calcium oreperation	166 144 98	16.5 14.3 9.7
**Number of 36-month follow-up TSH values at target** **0 (Group 1)** **1–2 (Group 1)** **3–5 (Group 2)** **≥ 6 (Group 3)**	188 527 775 260	10.7 30.1 44.2 14.8	**Age (Year)** 21–29 (*n* = 129) 30–39 (*n* = 267) 40–49 (*n* = 469) 50–59 (*n* = 434) 60–69 (*n* = 228) 70–79 (*n* = 70) >80 (*n* = 3)	**TSH values (mU/l)** 4.76 ± 8.48 3.47 ± 4.65 3.33 ± 4.85 3.86 ± 10.08 3.54 ± 9.69 3.09 ± 3.05 1.93 ± 0.57	

Patients in group 1 who had less ideal treatment target visits exhibited higher (i.e. worse) scores on a variety of scales, including goiter symptoms, hyperthyroidism and hypothyroidism symptoms, fatigue symptom scale, cognitive, anxiety, and depression scales, as well as social and sexual life disruption, impairment in daily life, cosmetic complaints as measured with the ThyPRO questionnaire. They also had lower scores on the Eq. 5D questionnaire than groups 2 and 3 (Table 4).

**Table 4:** The impact of personalized TSH target achievement rates on the ThyPRO and Eq. 5D questionnaires

Subscales	All patients	Group 1 (*n*:715)	Group 2 (*n*:775)	Group 3 (*n*:260)
**Goiter symptoms**	15.38 ± 16.99	17.23 ± 18.07 ^a. c^	14.33 ± 16.02	13.41 ± 16.30
**Hyperthyroid symptoms**	23.36 ± 18.27	25.0 ± 19.46 ^a^	21.67 ± 17.15	23.89 ± 17.74
**Hypothyroid symptoms**	31.06 ± 24.20	32.94 ± 24.51 ^a^	29.29 ± 23.75	31.15 ± 24.33
**Eye symptoms**	19.50 ± 19.57	19.70 ± 19.20	19.32 ± 20.21	19.49 ± 18.72
**Tiredness**	47.83 ± 16.25	48.84 ± 16.06 ^b^	46.89 ± 16.37	47.86 ± 16.31
**Cognitive problems**	30.79 ± 26.77	33.84 ± 28.22 ^a.c^	29.03 ± 25.64	27.63 ± 25.23
**Anxiety**	38.09 ± 25.81	40.18 ± 25.23 ^a^	36.37 ± 25.62	27.63 ± 25.23
**Depression**	42.04 ± 19.51	43.61 ± 20.19 ^a^	40.91 ± 19.37^d^	37.43 ± 26.12
**Emotional Susceptibility**	36.82 ± 18.40	38.38 ± 19.22 ^a^	35.55 ± 17.84	36.33 ± 17.50
**Impaired Social life**	15.95 ± 20.77	17.42 ± 21.68 ^b^	14.90 ± 20.19	15.02 ± 19.71
**Impaired daily life**	19.37 ± 23.52	21.82 ± 25.14 ^a.c^	17.87 ± 22.58	17.09 ± 20.98
**Impaired Sex life**	22.83 ± 31.70	25.12 ± 32.98 ^b^	21.04 ± 30.79	21.93 ± 30.50
**Cosmetics complaints**	20.40 ± 23.69	22.40 ± 24.36 ^b.c^	19.45 ± 23.66	17.74 ± 21.48
**Eq. 5D-VAS scale**	70.77 ± 17.56	69.14 ± 18.58 ^a.c^	71.72 ± 16.76	72.41 ± 16.69

^a^. Comparison of groups 1 and 2 *p* < 0.01, ^b^ Comparison of groups 1 and 2 < 0.05, ^c^ Comparison of groups 1 and 3 *p* < 0.01, ^d^ Comparison of groups 2 and 3. *p* < 0.05.

## Discussion

In thyroid hormone replacement therapy, an adequate assessment of thyroid function tests is critical for the success of the treatment process. Research on the status of LT-4 treatment success in the general population is inconclusive. Patients receiving thyroid hormone replacement may not achieve ideal TSH levels for several reasons. Not taking the medication regularly or following the recommended dietary changes can have a negative impact on treatment effectiveness. Other health problems or drug interactions can have an impact on thyroid hormone metabolism. Moreover, lifestyle factors including dietary habits, stress levels, and physical activity can influence thyroid hormone levels [3–13].

Regular endocrinology visits, patient education, assistance for taking medication, medication affordability and accessibility, number of medications used, busy work schedule are all effect the medication adherence [14]. In our study, patients had been receiving levothyroxine replacement therapy for approximately 10 years or more. Despite this, 40% of them had never reached their individual target TSH levels. Almost three quarters of the study group were not working and the number of shift workers was quite low but 33% of them were forgetting to take the drug. In a study, approximately 30% of participants had a medication possession ratio below 80% which means that at least 73 cumulative days without LT-4 intake in a year [15]. Most of our patients reported that they were careful about timing of drug intake and 73.3% of them were waiting for at least 30 min before breakfast. The drug has a relatively long half-life, which can be an advantage if it’s not taken for a day or two. Treatment success can increase by questioning the patients at each visit about whether they are using levothyroxine correctly and by repeating the patient education about the treatment principles.

Okosieme OE, et al. reported that 37.2% of 1037 levothyroxine users in the United Kingdom received inadequate treatment [16]. In a German study that regularly monitored TSH levels in patients receiving LT4 treatment, researchers found that 21.8% of patients receiving LT4 replacement did not meet the target TSH level. Additionally, 60% of patients had their TSH levels checked at least every 6–12 months, while the remaining patients had their levels checked over a longer period [17]. In another study of 1755 patients with primary hypothyroidism, it was found that despite LT4 treatment, approximately half of the hypothyroid patients had serum TSH values outside the reference range [18]. In the present study, 10.7% of patients never reached the target at 3-year follow-up. During the 3-year follow-up, an average of 6 visits revealed that 40.6% of the patients were on target less than three times. A comprehensive approach is required to achieve optimal TSH levels. According to the literature, multiple variables within each community have an impact on the efficacy of LT4 replacement therapy. As a result, more detailed information is needed about the relationships between thyroid hormone therapy sufficiency and various clinical characteristics (for example, age, gender, weight, duration of treatment, presence of comorbidities, and accessibility to healthcare).

Our findings showed that patients who met more TSH targets during the follow-up period had a higher disease-related quality of life. Furthermore, the ThyPRO scale revealed that patients who met fewer ideal TSH targets during the follow-up period had more negative responses in goiter, hyper/hypothyroidism, fatigue symptom, cognitive disorders, anxiety and depression status, social, sexual, and daily life impairment scales. Like our study, several studies demonstrated an improvement in quality of life especially on cognitive function upon achieving TSH targets [19, 20]. On the other hand, different studies have reported a decrease in quality of life for patients receiving LT4 replacement compared to the general population, regardless of TSH levels [21, 22]. In another study evaluating hypothyroid patients treated with levothyroxine demonstrated no significant differences in mental health or quality of life across different etiologies of hypothyroidism, while subtle variations in thyroid hormone status, particularly the FT3/FT4 ratio, were associated with psychological outcomes [23]. A meta-analysis of patients with subclinical hypothyroidism found that thyroid hormone therapy did not significantly improve overall quality of life, based on findings from 21 randomized clinical trials involving 2192 participants [24]. These findings suggest that thyroid problems involve not only hormonal balance, but also psychological and social factors, as well as distinct molecular pathways such as the function of deiodinases in various tissues. Likewise, a recent study found that the severity of hypothyroidism does not always correlate with quality of life in patients with newly diagnosed Hashimoto’s thyroiditis (REF), again suggesting that factors beyond TSH and peripheral thyroid hormone levels significantly influence patient well-being [25].

Especially adherence to treatment is challenging in elderly patients. Also polypharmacy is an important issue in these patients. Drug interactions may occur with other medications, absorption may be impaired, medications or dosages may be confusing for the patient. Treatment should be as simple as possible in older adults. The risk of cardiac morbidity and mortality, osteoporosis, cognitive dysfunction, and muscle deficiency increase in elderly patients. It is necessary to avoid over-treatment in older adults considering that the upper limit of target TSH is increased. In our study group, the mean TSH concentration of 129 patients aged 21–29 was 4.76 ± 8.48 mIU/L while the mean value for those aged 70–79 years was 3.09 ± 3.05 mIU/L. It should be remembered that there may be a need to reduce the levothyroxine dose as age increases.

In our study, patients who failed to achieve the target TSH level exhibited statistically significantly lower EQ-5D scores indicating a decline in health-related quality of life compared to patients with better long-term TSH control. Han and colleagues reported that higher TSH levels in untreated elderly patients were associated with better EQ-5D scores [26]. In untreated elderly individuals, elevated TSH levels may be part of an adaptive reduction in metabolic rate aimed at preserving energy balance and functional status; in contrast, fluctuations in TSH levels in patients receiving levothyroxine therapy may dynamically reflect an imbalance in exogenous hormone replacement. Such an imbalance may have led to intermittent hypo or hyperthyroidism at the tissue level, impaired peripheral hormone signaling, and a persistent symptom burden, ultimately resulting in a decline in quality of life. Rather than a single TSH measurement, the duration spent at a dynamically individualized optimal TSH level may better reflect quality of life.

It is also worth noting that the current study dataset is primarily based on population surveys and contains limited individual patient information. One of our study’s strengths is its broad representation, with 26 different centers. Including patients who have been followed at the same center for at least 3 years increases the reliability of the data. Another advantage of our research is that we investigated both overtreatment and undertreatment in a large population-based cohort. Examining the frequency of follow-up on variables over time is a method for which there is no clear data in the literature. The most significant limitation of our study is the lack of access to TSH levels before initiating levothyroxine treatment and inability to detect TSH fluctuations between visits. Also we did not have ThyPRO scale data from that period. Furthermore, the studied population’s comorbidities and health perceptions may differ from the general population. This may have reduced the results’ generalizability.

Achieving an individualized TSH target is important in replacement therapy for hypothyroidism, and achieving euthyroidism positively affects the patient’s quality of life and cognitive functions. Patients whose TSH concentrations remain at target for a long time have a higher quality of life.

## Data Availability

No datasets were generated or analysed during the current study.
